# Therapeutic potential of multifunctional myricetin for treatment of type 2 diabetes mellitus

**DOI:** 10.3389/fnut.2023.1175660

**Published:** 2023-05-26

**Authors:** Naomi Niisato, Yoshinori Marunaka

**Affiliations:** ^1^Department of Health and Sports Sciences, Faculty of Health and Medical Sciences, Kyoto University of Advanced Science, Kameoka, Japan; ^2^Medical Research Institute, Kyoto Industrial Health Association, Kyoto, Japan; ^3^Research Unit for Epithelial Physiology, Research Organization of Science and Technology, Ritsumeikan University, Kusatsu, Japan; ^4^Kyoto Prefectural University of Medicine Graduate School of Medical Science, Kyoto, Japan

**Keywords:** T2DM, GLP-1, myricetin, hyperglycemia, insulin resistance, flavonoid, pancreatic β-cell

## Abstract

Type 2 diabetes mellitus (T2DM) is a metabolic disorder characterized by chronic hyperglycemia, insulin resistance, and insufficient insulin secretion. It is considered that chronic hyperglycemia causes serious problems due to diabetic complications such as retinopathy, nephropathy, and neuropathy. Primarily, treatment in T2DM is pharmacologically tried by using drugs that are insulin sensitizers, insulin secretagogues, α-glucosidase inhibitors, and glucose transporter inhibitors. However, long-term application of these drugs frequently induces various harmful side effects, suggesting that the importance of taking advantage of natural products like phytochemicals. Accordingly, flavonoids, a group of phytochemicals, have attracted attention as components of natural products which are effective in the treatment of several diseases containing T2DM and are strongly recommended as food supplements to ameliorate T2DM-related complications. Several well-studied flavonoids such as quercetin and catechin are known to have anti-diabetic, anti-obesity, and anti-hypertensive actions, although a huge number of flavonoids are still under investigation and their actions are not fully understood. In this situation, myricetin is being shown to be a multiple bioactive compound to prevent and/or suppress hyperglycemia through inhibiting digestion and uptake of saccharides and enhancing insulin secretion as a possible GLP-1 receptor agonist, and to ameliorate T2DM-related complications by protecting endothelial cells from oxidative stress induced by hyperglycemia. In this review, we summarize the multiple effects of myricetin on the targets of T2DM treatment, comparing with different flavonoids.

## 1. Introduction

Type 2 Diabetes mellitus (T2DM) is a metabolic disorder mainly characterized by chronic hyperglycemia, insulin resistance, and insufficient insulin secretion. T2DM impairs the ability of insulin to lower blood glucose levels, resulting in the risk of micro-and macro-vascular complications such as diabetic retinopathy, diabetic neuropathy, diabetic nephropathy, and cardiovascular diseases due to the increased blood glucose, consequently decreased quality of life (1–3). These life-threatening complications make diabetes more severe than other diseases. Hence it is essential to maintain blood glucose levels as close to normal levels as possible for the management of T2DM. As traditional treatments for T2DM have focused on pancreatic *β*-cell dysfunction and insulin resistance, it is additionally recognized that hyper-glucagonemia in pancreatic α-cells with elevation of glucose absorption in the kidney (4) and tissue inflammation (5) are also associated with T2DM. In International Diabetes Federation (IDF) Atlas 2021, although the number of people with diabetes is estimated to 537 million worldwide in 2021, this number is expected to reach up to 643 million by 2030 and 783 million by 2045 that is serious health and economic problems. Therefore, beneficial and secure therapeutic approach is essentially required for eradication of T2DM.

**Figure 1:**
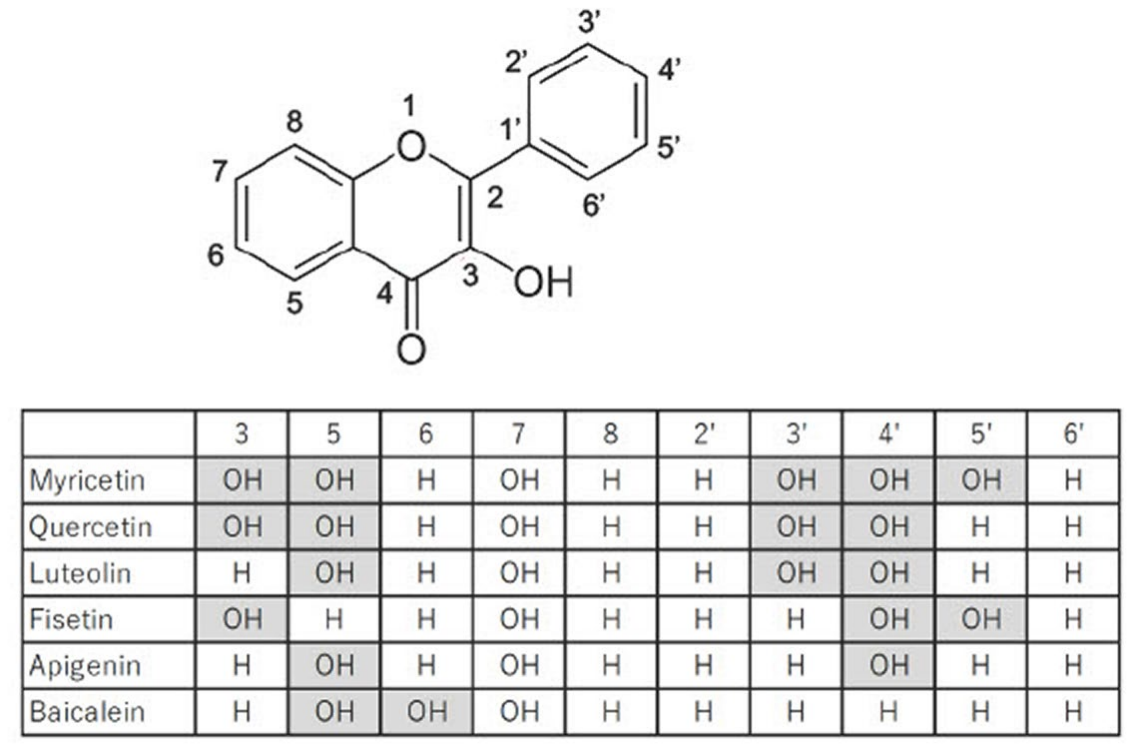
Structures of myricetin and related flavonoids.

Flavonoids are large family of compounds that possesses a common chemical structure (C6-C3-C6) and are divided into subclasses such as flavones, isoflavones, flavanones, flavanols, flavanes and flavonols structure-dependently (Figure 1). Flavonoids are basically pigment compositions that are synthesized by various plants for protection themselves from active oxygen generated on UV exposure and from harmful insects. In the past 20 years, flavonoids derived from plants have attracted a wide range of attention due to their various biological activities, including their anti-oxidant, anti-inflammatory, anti-tumor, anti-diabetic, anti-obesity, anti-hypertensive, and anti-viral actions (6–10). Previous studies provide numerous evidences that the flavonoids owing to their bioactive properties have possibilities of clinical application as well as effective nutraceuticals on treatment for T2DM. However, it is still unclear which flavonoid is more effective and safer for treatment of T2DM. Based on recent studies regarding efficacy of myricetin on treatment for T2DM, myricetin might be a candidate of clinical application for T2DM.

In this review, we focus on myricetin as a flavonol, which might have therapeutic potential to overcome T2DM and summarize its biological activities in compliance with therapeutic targets.

## 2. Inhibition of glucose intake with myricetin

Both carbohydrate digestibility and absorptivity are key determinants for regulation of postprandial hyperglycemia. One strategy for suppression of postprandial hyperglycemia is to restrict the activities of carbohydrate digestive enzymes such as α-glucosidase and α-amylase within gastrointestinal tract, resulting in the reduction of monosaccharide absorption rates. Indeed, α-glucosidase inhibitors, i.e., acarbose, voglibose and miglitol, have therapeutic effects on postprandial hyperglycemia (11) for treatment of T2DM. Since actual α-glucosidase inhibitors used in the control of T2DM have been associated with powerful gastrointestinal side effects, the exploration of novel, effective and safer compounds is considered as a hot topic of clinical research. Recently flavonoids, such as quercetin and myricetin, have exhibited promising anti-diabetic activities, including the inhibition of α-amylase and α-glucosidase (12, 13).

Inhibitory effects of flavonoids on *α*-glucosidase and α-amylase are determined with *in vitro* assay. The IC_50_ for acarbose, quercetin, myricetin, luteolin and baicalein to inhibit α-glucosidase are 1037.60 ± 189.88 μM, 10.92 ± 4.04 μM, 17.78 ± 1.75 μM, 42.36 ± 7.72 μM and 303.37 ± 57.19 μM, respectively. In contrast, the IC_50_ for acarbose, quercetin, myricetin, luteolin and baicalein to inhibit *α*-amylase are 0.83 ± 0.09 μM, 28.55 ± 1.41 μM, 28.78 ± 1.84 μM, 51.60 ± 4.93 μM and 1276.67 ± 106.54 μM, respectively (14). The inhibitory action of quercetin, myricetin and baicalein against α-glycosidase and α-amylase indicates the same rank order (i.e., quercetin > myricetin > baicalein) and acarbose is a more specific inhibitor for α-amylase than α-glucosidase. These results show that myricetin is a relatively strong inhibitor for α-amylase and α-glucosidase similar to quercetin.

To screen inhibitory effects of flavonoids against α-glucosidase, 11 flavonoids are determined by *in vitro* assay (15). For inhibition of α-glucosidase activity, IC_50_ against myricetin, myricetrin, dihydromyricetin, quercetin, catechin and gallocatechin are 40.7 ± 6.0 μg/mL, 98.5 ± 12.0 μg/mL, 65.7 ± 10.0 μg/mL, 108.0 ± 17.0 μg/mL, 1037.6 ± 189.9 μg/mL, and 1613.0 ± 138.0 μg/mL, respectively. The commercial α-glucosidase inhibitor, acarbose, is determined as 691.0 μg/mL. Therefore, the lower IC_50_ values of myricetin, myricetin derivatives and quercetin would have a therapeutic potential as an alternative to acarbose (15). Furthermore, the binding assay has demonstrated that myricetin, myricetrin, quercetin and catechin inhibits the α-glucosidase activity in a competitive mode, forming flavonoid-glucosidase complex at the enzyme active site, while gallocatechin inhibits the enzyme in an uncompetitive one (15). For myricetin, the docking study confirms the essential role of C4 = O, 3-OH and 5′-OH in *α*-glucosidase inhibition. Glycosylation of myricetin (switching myricetin to myricetrin) changes the binding conformation with the three hydrogen bindings disappeared that decreased the inhibitor activity (15) (Figure 1). Accumulating information on relationship between structure and inhibitory activity suggests that flavonoids have the optimal structure to inhibit α-glucosidase.

Moreover, 27 dietary flavonoids that act as α-glucosidase inhibitors are chosen and comparatively studied. *In vitro* experiments of α-glucosidase inhibition have shown that myricetin (IC_50_ = 11.63 ± 0.36 μM) possesses the strongest inhibitory effect, followed by apigenin-7-*O*-glucoside (IC_50_ = 22.80 ± 0.24 μM) and fisetin (IC_50_ = 46.39 ± 0.34 μM) (16). Then, the extracts from species of *Myrcia* containing 3-O-rhamnoside derivatives of myricetin, quercetin and gallic acid as the major constituents inhibit 90–500 times more α-glucosidase than acarbose and display a mild inhibition against α-amylase (17). On the other hand, Yao et al. have reported that myricetin intake (120.5 ± 95.7 mg/day with apple, peach, orange, pineapple, and sweet potato being the main food sources) is inversely related to the prevalence of T2DM in this Chinese population, suggesting a protective effect of myricetin in the development of T2DM by their designed cross-sectional population study, which includes 24,138 subjects, with 1,357 of them diagnosed with T2DM (18). As far as we know, this study might be the first large-scale study to investigate the association between the intake of the single flavonoid substance, myricetin, and the prevalence of T2DM in a Chinese population, suggesting the importance of dietary intake of flavonoids for slow progress or treatment of T2DM.

## 3. Inhibition of glucose transport with myricetin

The activation of glucose transport system across the plasma membrane in response to insulin is a central mechanism in the regulation of glucose homeostasis. In the treatment of T2DM, it is considered that management of blood glucose level through regulation of glucose transporters such as glucose transporter 2 (GLUT2), sodium-dependent glucose transporter 2 (SGLT2) (19) and GLUT4 is also effective to its amelioration. After digestion of food, absorbed glucose molecules are passively diffused into the pancreatic β-cell through GLUT-2 that is a trigger for insulin secretion. Previous works strongly suggest that inhibition of GLUT-2 obviously suppresses glucose-stimulated insulin secretion in the pancreatic *β*-cells (20, 21).

The streptozotocin (STZ) cadmium (Cd) induced diabetic nephrotoxic rats (STZ-Cd rats) show significant increases of plasma glucose, glycated hemoglobin, glyconeogenesis and glycogenolysis enzymes, renal function markers (i.e., creatinine and urinary albumin) and significant decreases of plasma insulin, glycogen and glycogen synthase with insulin signaling molecule expression. The administration of myricetin to the STZ-Cd rats significantly normalizes the blood glucose level, the activities of carbohydrate metabolic enzymes and renal function markers and insulin dependent protein expression such as GLUT-2, GLUT-4 and IRS-1, suggesting that myricetin remarkably improves carbohydrate metabolism and glucose transport, subsequently enhancing glucose uptake into cells, glucose utilization and renal function in STZ-Cd rats (22). These results demonstrate that myricetin clearly ameliorated symptoms of T2DM in the liver, the skeletal muscle, the pancreas, and the kidney as target organs.

As the digested carbohydrate, glucose, is mainly absorbed through apical GLUT2 in addition to SGLT1 and GLUT5 into small intestinal cells, inhibition of GLUT2 is a strategy for suppression of hyperglycemia in T2DM patients. To evaluate flavonoids actions including myricetin and quercetin on the apical GLUT, effect of flavonoids on uptake of fructose and 2-deoxy glucose (2DG) has been studied in GLUT2 expressed *Xenopus* oocytes. The IC_50_ in fructose assay for quercetin, myricetin, fisetin, and luteolin are 15.9 μM, 11.9 μM, 42.2 μM and 22.7 μM, respectively. On the other hand, the IC_50_ in 2DG assay for quercetin, myricetin, fisetin, luteolin are 12.7 μM, 17.2 μM, 47.2 μM and 30.4 μM, respectively (23). These experiments have shown that myricetin is a powerful inhibitor for intestinal GLUT2, leading to therapeutic potential of myricetin for treatment of hyperglycemia through suppression of intestinal glucose absorption. Another strategy to ameliorate hyperglycemia is to enhance uptake of blood glucose by elevating expression and/or activity of GLUT4. Myricetin has been reported to have a hypoglycemic activity in high fat diet (HFD) fed STZ-induced diabetic rats (24). The continuous administration of myricetin in HFD/STZ rat is reported to significantly reduce serum glucose and insulin through increasing expression of insulin receptor and GLUT4 genes. Furthermore, myricetin protects pancreatic cells from HFD/STZ-induced apoptosis through regulation of Bax and Bcl-2 genes (24). It has been established that rats fed a high-fructose diet induce insulin resistance, hyperinsulinemia, hypertriglyceridemia and hyperglycemia, and would be a model animal for T2DM (25, 26). In fructose chow-fed rats, the intravenous injection of myricetin (1 mg/kg per injection, 3 times daily) for 14 days markedly reduces the hyperglycemia and hypertriglyceridemia (27). The treatment with myricetin also improves insulin sensitivity through the enhancement of insulin action on IRS-1-associated PI3-kinase/Akt activity and GLUT 4 surface expression in soleus muscles in fructose chow-fed rats (27). These experimental results suggest that myricetin ameliorates hyperglycemia in T2DM through inhibition of GLUT2 in the intestine and enhancement of GLUT4 surface expression and/or activity in the muscles, and that anti-diabetic action of myricetin is relatively potent compared to other types of flavonoids such as quercetin, fisetin and luteolin.

## 4. Myricetin as a GLP-1R agonist

Glucagon-like peptide-1(GLP-1) has been discovered in 1990 as a gut hormone that is released from intestinal L cell after oral glucose administration (28). The released GLP-1 binds to GLP-1 receptor (GLP-1R) in pancreatic β-cells and stimulates the secretion of insulin in a blood glucose level-dependent manner to control blood glucose levels within the normal range. In comparison to the direct administration of insulin, GLP-1 has advantage to avoid hypoglycemia because GLP-1 is released depending upon the increased blood glucose level. However, GLP-1 has poor stability due to its degradation with dipeptidyl peptidase 4 (DPP-4). GLP-1 has been indicated to ameliorate blood glucose levels primarily through mechanisms that promote insulin secretion and suppress glucagon secretion. In addition, GLP-1 protects β-cells from apoptosis by oxidative stress. The physiologic properties of GLP-1 make its stable agonist a potent candidate for a novel drug target in the treatment of T2DM. Actually, exendin 4 (an agonist for GLP-1R) is clinically applied and widely used for treatment of T2DM, however exendin 4 has gastrointestinal side effects containing malevolence and vomition.

In treatment of T2DM, myricetin has been demonstrated to reduce plasma glucose levels (29, 30), to protect pancreatic cells and to restore islet function. Myricetin also attenuates the hyperglycemia in STZ-induced diabetic rats as with previous reports (31). Li et al. (32) have reported that *in vitro* binding assay shows dose-dependent binding of myricetin to GLP-1R and that myricetin failed to exert its glucoregulatory in GLP-1R knockout (KO) mice, strongly demonstrating that myricetin is an orally active, small-molecule GLP-1R agonist. Furthermore, administration of exendin 9–39 (an antagonist of GLP-1R) with myricetin dose-dependently suppresses the anti-diabetic actions of myricetin (33), suggesting that myricetin might be identified as an agonist of GLP-1R, such as exenatide, liraglutide, and albiglutide. This property of myricetin as a GLP-1 agonist is unique among numerous flavonoids as far as we know, in addition to the actions on regulating glucose transport through GLUT2 and GLUT4.

The proteolytic enzyme, DPP4, is expressed in the most of main organs. The DPP-4 expression is upregulated in T2DM which is associated with the rapid degradation of GLP-1 (34). It is considered that DPP-4 inhibitors prevent diabetes-induced activation of node like receptor family, pyrin domain containing 3 (NLRP3) inflammasome that causes metabolic inflammation and insulin resistance (35, 36). In STZ-induced diabetic Wistar rats, myricetin decreases DPP-4 activity, its expression, and diabetes-induced NLRP3 inflammasome activation, increasing circulating GLP-1 and insulin levels (30). This suggests that myricetin would verify to be a promising inhibitor of DPP-4 *in vitro* and *in vivo* and improve the symptoms of T2DM (30). Ultimately, it is demonstrated that myricetin activates GLP-1R to stimulate insulin secretion and to attenuate glucagon secretion, and that myricetin enhances native GLP-1 by inhibiting DPP-4.

## 5. Inhibitory potential of myricetin for IAPP aggregation

T2DM is characterized as a metabolic disorder with defects in both insulin secretion and insulin action, and is also characterized by the presence of fibrillar amyloid deposits in the pancreatic islet of Langerhans, suggesting that T2DM is classified to protein-misfolding disease. Amyloid deposits are known to be correlated to other diseases such as Alzheimer’s disease, Parkinson’s disease, and spongiform encephalopathy in addition to T2DM. In 1987, two research groups with Westermark and Cooper (37, 38) independently discovered that the main component of islet amyloid is a 37-amino acids polypeptide named islet amyloid polypeptide (IAPP). Human IAPP (hIAPP) is the major component of the amyloid deposits found in the pancreatic islets of patients with T2DM. The aggregation of IAPP is believed to play a direct role in the death of pancreatic β-cells in T2DM. Preventing the initial aggregation event of IAPP is one strategy for slowing or attenuating the progression of this disease. Recently, several flavonoids have been shown to inhibit IAPP aggregation (39, 40). To elucidate an inhibitory potential of myricetin for IAPP aggregation, thioflavin T binding assay has been performed. As a result, myricetin prevents IAPP aggregation in a dose-dependently (41). Moreover, myricetin protects living cells (PC12 cells) from IAPP-induced cell death detected by MTT assay. These experimental results indicate that myricetin is a strong inhibitor of IAPP aggregation and has a potential for the development of an amyloid inhibiting therapy. In a previous study, myricetin supplementation inhibits hIAPP aggregation and disaggregates preformed fibrils into non-toxic species. This protection is accompanied by inhibition of oxidative stress, reduction in the lipid peroxidation and the associated membrane damage, and restoration of mitochondrial membrane potential in INS-1E cells (42). However, at the present only a few studies have been reported on myricetin’s ability to inhibit IAPP aggregation. Therefore, the mechanism of myricetin’s action needs to be further investigated.

## 6. Miscellaneous actions of myricetin

Endothelial dysfunction might be recognized as the initial stage for cardiovascular disease, a complication of diabetes. Generally, exposure of endothelial cells to high glucose (HG) environments elicits HG-induced apoptosis through oxidative damage. Pretreatment of myricetin can restore the viability of endothelial cells under oxidative stress in umbilical vein endothelial cells. In addition to prevention of apoptosis, myricetin reduces the HG-induced increase in lipid peroxidation levels, Bax and Bcl-2 expression levels, cleaved caspase-3 levels, suggesting that myricetin significantly lowers the risk of T2DM progression through protection of endothelial cells (43). Further, the study to evaluate effect of myricetin on protection against diabetic osteoporosis in STZ-induced diabetic rats shows that myricetin could effectively improve abnormal bone metabolism which may provide beneficial medicine on diabetic bone disease (44). Treatment with myricetin (100 mg/kg/day) for 12 weeks significantly ameliorates bone mineral density, alkaline phosphatase, osteocalcin, trabecular bone microarchitecture, oxidative damage, superoxide dismutase (SOD) and catalase activity. On the other hand, diabetic peripheral neuropathy (DNP) is considered as one of serious complications of diabetes mellitus. In previous studies, myricetin has been proved to possess neuroactive and antioxidative actions. Another report has indicated that myricetin ameliorates diabetes-induced impairment in sensation, nerve conduction velocities, and nerve blood flow. In addition, myricetin suppresses generation of advanced glycation end-products (AGEs) and reactive oxygen species (ROS), and elevates the Na^+^, K^+^-ATPase activity and anti-oxidant activities in nerves of diabetic rats. These experimental results suggest that myricetin could restore the impaired motor and sensory functions under diabetic conditions (45). In recent years, as numerous studies have indicated that there is a relationship between T2DM and the intestinal flora, the gut microbiota might be a new therapeutic target for treatment of T2DM and its related diseases. The study to evaluate effect of myricetin on intestinal flora in diabetic model mice have demonstrated that myricetin improved microflora as a signature of T2DM (46). Further, myricetin significantly ameliorated polydipsia, polyphagia, polyuria, and weight loss in T2DM mice. Thus, myricetin is a multifunctional compound which has ability to improve T2DM-induced various symptoms such as prevention of endothelial cells from apoptosis by oxidative stress, suppression of osteoporosis, restoration of neuronal functions, and disorders in the intestinal flora.

## 7. Conclusion

**Figure 2:**
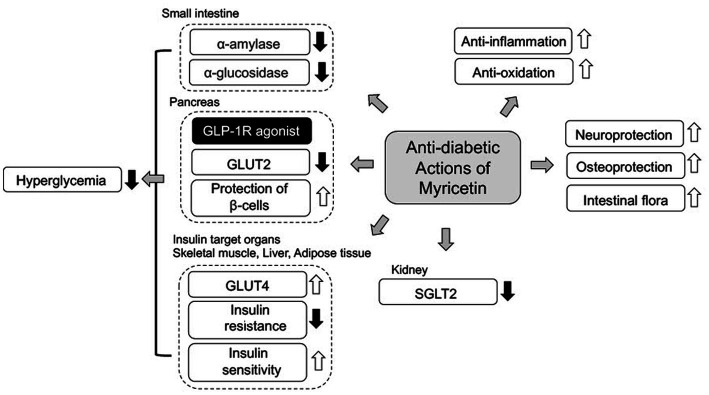
A scheme of anti-diabetic mechanism of myricetin. Myricetin mainly ameliorates hyperglycemia in T2DM with 1) inhibition of carbohydrate digestion by α-glucosidase and α-amylase, 2) inhibition of glucose transport through GLUT2 and SGL2, 3) action as GLP-1R agonist with stimulation of insulin secretion. Additionally, myricetin ameliorates T2DM complications through anti-oxidative, anti-inflammatory and neuroprotective actions.

In the past, numerous flavonoids and their derivatives have been elucidated to understand their biological actions and various functions as therapeutic targets for treatment of T2DM. This review focuses on the therapeutic potential of anti-diabetic actions on myricetin for treatment of T2DM (Figure 2). As noted above, anti-diabetic actions of myricetin are summarized as 1) inhibitors of α-glucosidase and α-amylase for prevention of carbohydrate digestion, 2) inhibitors of GLUT2 for suppression of glucose absorption through a specific transporter, 3) elevation of GLUT4 expression for enhancement of insulin-dependent glucose uptake into the target organs, 4) an agonist for GLP-1R to enhance insulin secretion, suppression of glucagon secretion, and protection of pancreatic β-cells, 5) DPP-4 inhibitors for prevention of GLP-1 degradation, and 6) prevention of hIAPP aggregation and NLRP3 activation for protection of pancreatic β-cell, 7) protection of endothelial cells from HG-induced apoptosis, 8) protection against diabetic osteoporosis, 10) prevention of DNP through suppression of oxidative stress and 11) improvement of the intestinal flora. It is specially mentioned that 10 or more anti-diabetic actions coexist in a flavonoid, myricetin (Figure 2). Some of them, actions as a GLP-1R agonist and a DPP-4 inhibitor, seem to be prominently useful for clinical application with further investigation. However, there are several issues that need to be clarified for clinical application of myricetin such as structural and chemical information for its appropriate solubility, permeability, absorbing rate, and stability in our body (47). At least, active dietary intake of myricetin from vegetables and fruits has suppressive effects on hyperglycemia. Flavonoids have recently attracted attention as source materials for development of new anti-diabetic drugs. Accumulated evidence from various epidemiological, animal and *in vitro* studies have confirmed the beneficial effects of many dietary flavonoids containing myricetin in the treatment and management of T2DM and its related complications.

## Author contributions

NN and YM contributed to conception, constitution and contents of this review. NN mainly wrote the first draft of this manuscript. All authors contributed to the article and approved the submitted version.

## Funding

This work was funded by Grants-in-Aid for Scientific Research (B) from the Japan Society of the Promotion of Science (JSPS KAKENHI Grant Number JP18H03182 and JP21H03368) to YM.

## Conflict of interest

The authors declare that the research was conducted in the absence of any commercial or financial relationships that could be construed as a potential conflict of interest.

## Publisher’s note

All claims expressed in this article are solely those of the authors and do not necessarily represent those of their affiliated organizations, or those of the publisher, the editors and the reviewers. Any product that may be evaluated in this article, or claim that may be made by its manufacturer, is not guaranteed or endorsed by the publisher.
